# Unconscious thought and deliberation without attention: A miracle or a mirage?

**DOI:** 10.1007/s40037-014-0127-y

**Published:** 2014-07-01

**Authors:** Eugène J. F. M. Custers

**Affiliations:** School of Medical Science, Center for Research and Development of Education, University Medical Center Utrecht, HB Building 4.05, PO Box 85500, 3508 GA Utrecht, the Netherlands

**Keywords:** Research in medical education, Reflection/critical thinking, Deliberation without attention hypothesis, Unconscious thought theory

Since the time of Sigmund Freud (1856–1939), people have been fascinated by the possibility of a powerful and influential ‘unconscious’ that controls much of our behaviour. Though Freud conceives of this unconscious largely in terms of emotional urges and powers to suppress these urges, a more cognitive interpretation in terms of mental processes is highly feasible [1, 2]. Most of us will probably occasionally have the experience of suddenly ‘seeing’ the solution to a problem, or suddenly ‘knowing what to choose,’ after having been distracted for a while by other concerns. It is tempting to conclude from such experiences that, while we were distracted, some form of unconscious processing of the problem has continued which then resulted in the correct solution or choice ‘popping up’ in our consciousness. Basically, this was the point of departure for Dijksterhuis to develop his unconscious thought theory and the associated notion of ‘deliberation without attention’ [3–5]. According to this theory, unconscious thought leads to better performance, in particular in complex tasks, than conscious thought. The question is, how credible is this view?

To begin with, as Bonke et al. [6] aptly note, Dijksterhuis et al.’s unconscious thought theory diverges from mainstream views of human (un)consciousness, i.e. the dual system theories [7–9] which recently became popular among a much broader audience after the release of Daniel Kahneman’s 2011 book *Thinking, fast and slow* [10]. These theories distinguish two processing systems: one that operates fast, automatically, holistically, intuitively, and unconsciously (‘System 1’) and one that operates slowly, analytically, controlled, and consciously (‘System 2’). In this framework there is no room for deliberation without attention, as this would be contradictory to the fundamentals of the human mind. Neither Dijksterhuis and colleagues, nor proponents of the dual systems view, make any effort to align their views; in fact, they almost completely ignore each other’s work.

Some studies and discussion papers have been published in which the dual systems view has been applied to medical education, in particular to medical errors [11–13]. As yet, the Bonke et al. study [6] is the third to test the other view, deliberation without attention, in the medical domain. The two earlier studies pitted the quality of medical diagnosis after participants were engaged in either conscious thought or unconscious thought [14, 15]. The results were mixed. If the current Bonke et al. study [6] is added, the preponderance of the evidence would be against a beneficial effect of deliberation without attention, which is largely in line with findings outside the medical domain. I will not further discuss the conflicting experimental evidence; for the purpose of this commentary, it will be safe to say that the jury is still out.

In the remainder of this commentary I will discuss a question that I believe to be more interesting than detailed discussion of mixed outcomes, that is, *why* would unconscious thought be superior to conscious thought in making complex decisions? Dijksterhuis and colleagues [3, 4] provide two reasons: First, the capacity of unconscious thought is much larger than conscious thought. Indeed, few will contest that conscious thinking has limited capacity. Yet, this capacity can easily be extended by using artefacts, such as paper and pencil and, more recently, computers, iPads, and smart phones. Remarkably, this issue is not discussed by either Dijksterhuis and colleagues or by Bonke et al. In these studies, participants are prevented from taking notes, but they do not provide any justification for denying participants the use of such aids, in particular if they are asked to solve difficult problems. By asking participants to remember a substantial amount of information, the results of the Dijksterhuis et al. studies are not so much due to unconscious thought yielding superior decisions, but to conscious thought being put at a disadvantage—in other words, the ‘deck is stacked’ against conscious thinking. In fact, it might even be argued that conscious thinking—in general—is superior to unconscious thinking *because* there are many ways to circumvent its limited capacity. This remark has been made by others—e.g., Lassiter et al. [16] and Shanks [17] proposed memory limitations of conscious thinkers as the source of the apparent superiority of deliberation without attention. Yet, Dijksterhuis et al. [18] reject this suggestion, which seems incongruent, for capacity limitations of consciousness *are* in essence memory limitations.

Second, Dijksterhuis et al. [4] also claim that conscious thought can lead to suboptimal weighting of the importance of attributes or features of different choice alternatives (cars, apartments, holiday destinations, etc.). Unconscious thought, they claim, works ‘bottom up’ and weights the relative importance of different attributes of objects in a relatively objective and ‘natural’ way. But how would the unconscious be able to achieve this? In any case, the use of the word ‘weights’ suggests the ability to assign quantitative values to attributes and to combine these values into a judgment or decision. If this were true, it would have major consequences for medicine, in particular for medical diagnosis. In complex cases, physicians will often need to weigh numerous bits of evidence in order to arrive at a (differential) diagnosis, a task that is hard and takes effort. Wouldn’t it be much more efficient—and agreeable—to lean back for a few minutes and let your unconscious do the work, as Dijksterhuis and colleagues would recommend? Have you ever tried? In fact, another incongruence becomes apparent here. Dijksterhuis’ participants are asked to choose the best option according to a simple normative standard: the option with the highest number of positive attributes. No weighting of any kind is involved. But participants are not informed in advance about this; rather, the instructions are to choose the best (or most favourite) option, or to form an impression of the options. According to the Cognitive Continuum Theory [19, 20] there is a mismatch here between the participants’ approach (choosing the subjectively best option, forming an impression) and the optimal approach (counting positive and negative attributes). Suppose participants are, in advance, provided with the rules that determine the best choice, are allowed to use aids and to take as much time as they need: how are they expected to perform? Pretty close to perfect, one might guess. At least, much better than by using deliberation without attention, which resulted in a mere 60 % correct choices, not even taking into account the 25 % chance level [5]. Ironically, the conclusion would be that Dijksterhuis and colleagues test the effect of deliberation without attention with a type of task that is, given the proper instructions and facilities, ideally suited for *conscious* decision making.

To sum up, even if deliberation without attention were to ‘work’ within the constraints of the Dijksterhuis’ research paradigm, its practical relevance would be limited. If we can exploit consciousness’ full powers by allowing it to use all available aids, it will almost always provide better results than deliberation without attention. The only exception might be decisions in which preferences and post-choice satisfaction play a large role. For such problems, there is no normative standard; someone’s preference or satisfaction *is* the standard. Conscious thought might make the person more aware of the disadvantages of any particular choice, leading to weaker preferences or less post-choice satisfaction than after deliberation without attention. In practice, this could have ramifications for situations in which patients or students are allowed to choose between, e.g., different treatment options or different courses. In these circumstances, recommending not to think too much about the options might make sense, at least if the aim is to maximize the chooser’s satisfaction.
